# Periodontitis, Dental Procedures, and Young-Onset Cryptogenic Stroke

**DOI:** 10.1177/00220345241232406

**Published:** 2024-04-16

**Authors:** J. Leskelä, J. Putaala, N. Martinez-Majander, L. Tulkki, M. Manzoor, S. Zaric, P. Ylikotila, R. Lautamäki, A. Saraste, S. Suihko, E. Könönen, J. Sinisalo, P.J. Pussinen, S. Paju

**Affiliations:** 1 Department of Oral and Maxillofacial Diseases, University of Helsinki, Helsinki, Finland; 2 Neurology, Helsinki University Hospital and University of Helsinki, Helsinki, Finland; 3 Centre for Host-Microbiome Interactions, Faculty of Dentistry, Oral & Craniofacial Sciences, King’s College London, London, UK; 4 Neurocenter, Turku University Hospital, University of Turku, Turku, Finland; 5 Heart Centre, Turku University Hospital, University of Turku, Turku, Finland; 6 Department of Medicine, Division of Cardiology, Helsinki University Central Hospital, Helsinki, Finland; 7 Institute of Dentistry, University of Turku, Turku, Finland; 8 School of Medicine, Institute of Dentistry, University of Eastern Finland, Kuopio, Finland

**Keywords:** endotoxemia, dental treatment, thrombosis, embolism, cardiovascular disease, ischemic stroke

## Abstract

Periodontitis is associated with an increased risk of ischemic stroke, and the risk may be particularly high among young people with unexplained stroke etiology. Thus, we investigated in a case-control study whether periodontitis or recent invasive dental treatments are associated with young-onset cryptogenic ischemic stroke (CIS). We enrolled participants from a multicenter case-control SECRETO study including adults aged 18 to 49 y presenting with an imaging-positive first-ever CIS and stroke-free age- and sex-matched controls. Thorough clinical and radiographic oral examination was performed. Furthermore, we measured serum lipopolysaccharide (LPS) and lipotechoic acid (LTA) levels. Multivariate conditional regression models were adjusted for stroke risk factors, regular dentist visits, and patent foramen ovale (PFO) status. We enrolled 146 case-control pairs (median age 41.9 y; 58.2% males). Periodontitis was diagnosed in 27.5% of CIS patients and 20.1% of controls (*P* < 0.001). In the fully adjusted models, CIS was associated with high periodontal inflammation burden (odds ratio [OR], 95% confidence interval) with an OR of 10.48 (3.18–34.5) and severe periodontitis with an OR of 7.48 (1.24–44.9). Stroke severity increased with the severity of periodontitis, having an OR of 6.43 (1.87–23.0) in stage III to IV, grade C. Invasive dental treatments performed within 3 mo prestroke were associated with CIS, with an OR of 2.54 (1.01–6.39). Association between CIS and invasive dental treatments was especially strong among those with PFO showing an OR of 6.26 (1.72–40.2). LPS/LTA did not differ between CIS patients and controls but displayed an increasing trend with periodontitis severity. Periodontitis and recent invasive dental procedures were associated with CIS after controlling for multiple confounders. However, the role of bacteremia as a mediator of this risk was not confirmed.

## Introduction

Stroke remains the second leading cause of death globally (Feigin et al. 2021). Strikingly, the incidence and prevalence of ischemic stroke have been increasing in the younger population during past decades (Scott et al. 2022). The most important modifiable risk factors for young adults are closely lifestyle related, including physical inactivity, smoking, and obesity, albeit established hypertension and diabetes are also important contributors (Aigner et al. 2017; Kivioja et al. 2018). Contrarily, lipid levels do not seem to play a significant role in young-onset stroke, in particular with regards to cryptogenic ischemic stroke (CIS) (Aigner et al. 2017; Martinez-Majander et al. 2021).

Prior studies have suggested periodontitis as an independent risk factor for ischemic stroke (Grau et al. 2004). Recently, a large Taiwanese retrospective cohort study showed that a registered periodontitis diagnosis is associated with an increased risk of developing a transient ischemic attack or a minor ischemic stroke in young adults, suggesting a stronger association for the age group of 20- to 40-y-olds than for 41- to 53-y-olds (Lee et al. 2022). Furthermore, compared to older ones, younger patients with ischemic stroke exhibit a larger proportion of strokes with unknown etiology, that is, they are labeled as CIS even after thorough and timely clinical examination (Martinez-Majander et al. 2021). Recent evidence also suggests that particularly individuals suffering from CIS and without traditional vascular risk factors could be the largest share of patients that make up the increasing incidence of young-onset ischemic strokes (Li et al. 2022).

Virulence factors of bacteria have been a potential study target when searching for potential mediators and determinants for the association between ischemic stroke and periodontitis. These include lipopolysaccharides (LPS, or endotoxin) and lipotechoic acid (LTA), located in the outer membrane of Gram-negative bacteria and the cell wall of Gram-positive bacteria, respectively. The main route for LPS passage into the circulation, endotoxemia, is considered to be from the gut (Chidambaram et al. 2022), while oral mucous and inflamed gingival tissues play a conjoint role (Geerts et al. 2002; Pussinen et al. 2022). Studies have shown conflicting results regarding the relationship between endotoxemia and ischemic stroke, with some studies in support (Hakoupian et al. 2021; Leskelä et al. 2021; Zheng et al. 2022) and some suggesting no association (Leskelä et al. 2020). Knowledge of the circulating LTA and its association with stroke is limited (Hakoupian et al. 2021).

Dental procedures are known to cause at least transient bacteremia (Forner et al. 2006; Lockhart et al. 2008) and a prothrombotic state (D’Aiuto et al. 2007; Tonetti et al. 2007), but the clinical relevance has remained debatable. Invasive dental treatments trigger a short-term acute-phase response (D’Aiuto et al. 2007), which may result in an increased risk for acute cardiovascular events (Elkind et al. 2020). In a recent systematic review, however, invasive dental treatments were not significantly associated with an increased risk of ischemic stroke, but the analyses were limited by a high level of heterogeneity in the published studies (Luthra et al. 2022).

We investigated whether periodontitis and recent invasive dental procedures are associated with the risk of young-onset cryptogenic ischemic stroke in an age- and sex-matched case-control study. We further hypothesized that bacteremia (i.e., circulating LPS and LTA) deriving from oral origin due to periodontitis or invasive dental operations mediates the association.

## Materials and Methods

### Study Participants and Recruitment

The present study, SECRETO Oral, is a substudy of the international, multicenter SECRETO (Searching for Explanations for Cryptogenic Stroke in the Young: Revealing the Etiology, Triggers, and Outcome; NCT01934725) study enrolling young adults (aged 18–49 y) presenting with an imaging-positive first-ever acute CIS (Putaala et al. 2017).

CIS patients were included after standardized diagnostic procedures (brain magnetic resonance imaging, angiography of intracranial and extracranial vessels, echocardiography, and screening for coagulopathies). CIS was defined according to the ASCO classification (Amarenco et al. 2009) with a few adaptations (Putaala et al. 2017). Stroke-free controls (1:1) were identified from population registry and matched with age and sex.

All enrolled participants gave written informed consent prior to inclusion in the study. The study complied with the guidelines of the Declaration of Helsinki and the manuscript with the Strengthening the Reporting of Observational Studies in Epidemiology guidelines. The study was approved by the ethics committee of the Helsinki and Uusimaa Hospital district.

Participants for SECRETO Oral were recruited between December 2013 and November 2019 at Helsinki University Hospital and Turku University Central Hospital.

### Clinical Oral Examination

Panoramic tomographs were taken from all CIS patients during the hospitalization period shortly after the incident and from the controls at the time of recruitment. An oral examination was scheduled for CIS patients and controls 8 to 12 wk after the end of the hospitalization period, depending on their rehabilitation. All oral examinations were carried out single-blinded by the same periodontal specialist (S. Paju) to avoid interexaminer differences. Periodontitis stage and grade were defined according to the 2017 proposition (Tonetti et al. 2018). To construct a summary variable to reflect the inflammatory burden posed by periodontitis, we calculated the Periodontal Inflammation Burden Index (PIBI) (Lindy et al. 2008) with the following formula: PIBI = [number of 4- to 5-mm probing pocket depth (PPD)] + 2 × [number of ≥6-mm PPD].

### Statistical Analysis

For power calculations, the prevalence of PPD ≥4 mm was used as the main variable of interest differentiating CIS patients from controls. With an alpha of 0.05, a power of 0.80, and an assumed prevalence of 55% in the control population (Aromaa et al. 2004), the minimum detectable odds ratio (OR) was 2.0.

All statistical analyses were performed using R software (version 4.0.5). Variables were expressed as medians with interquartile range (IQR) or counts with percentage. Univariate comparisons used the Wilcoxon matched pairs signed rank test and McNemar test. Pearson’s χ^2^ test was used in subgroup analysis. The Jonckheere–Terpstra test was used for trend testing, using 100,000 permutations for *P* value estimation.

To estimate the OR with a 95% confidence interval (CI), we constructed several multivariate models using conditional logistic regression with an increasing number of covariates: the crude model (model 1) was adjusted for age; model 2 was additionally adjusted for waist-to-hip ratio, heavy alcohol consumption, smoking, and patent foramen ovale (PFO). Model 3 (fully adjusted) included education level, regular dentist visits, and hypertension status in addition to all covariates in model 2. Interactions with PFO were analyzed in the model 3.

See Appendix methods for further details.

## Results

### Characteristics of the Study Population

Altogether, 348 participants (184 cases and 164 controls) were enrolled in SECRETO Oral, of whom 329 (169 cases and 160 controls) participated in the clinical oral examination (Table 1). After excluding participants without a matched pair, 146 case-control pairs were included in the analysis. The median time between CIS (cases) or recruitment to the main study (controls) and oral examination was 111 d (IQR, 92–139.8) and 66 d (72–133.5), respectively. The median time difference between CIS and blood sampling was 9 d (IQR, 7–13).

**Table 1.** Basic Clinical Characteristics of the Study Population.

Characteristic	CIS Patients (*n* = 146)	Controls (*n* = 146)	*P* Value^a^
Male sex	85 (58.2)	85 (58.2)	
Age, y	41.2 (35.7–46.1)	42.1 (35.2–46.5)	0.233
Education			**<0.001**
Primary or secondary education	79 (54.1)	49 (34.5)	
Higher education	67 (45.9)	93 (65.5)	
Obesity	78 (53.4)	69 (47.3)	0.308
Smoking, ever	72 (49.3)	61 (41.8)	0.229
Heavy alcohol consumption	36 (26.5)	20 (14.2)	0.055
Hypertension	38 (26)	25 (17.1)	0.093
Diabetes mellitus	7 (4.8)	6 (4.1)	1.000
Coronary heart disease	0 (0.0)	1 (0.7%)	1.000
Myocardial infarction	0 (0.0)	0 (0.0)	1.000
Regular dentist visits	77 (52.7)	92 (63.0)	0.101
Patent foramen ovale	101 (69.2)	56 (39.4)	**<0.001**
Stroke severity, NIHSS score			
Mild (NIHSS 0-4)	124 (84.9)	—	
Moderate to severe (NIHSS ≥5)	18 (12.3)	—	

Data expressed as median (interquartile range) or *n* (%).

CIS, cryptogenic ischemic stroke; NIHSS, National Institutes of Health Stroke Scale.

a P < 0.05 are bolded.

CIS patients had a significantly lower education level, had more hypertension, and more commonly exhibited heavy alcohol consumption compared to controls. PFO was more common among CIS patients compared to controls (*P* < 0.001).

At the time of the oral examination, CIS patients more often used antithrombotic or statin medications, whereas the difference in antihypertensive medications was nonsignificant (Table 2). Recent antibiotic treatments for any indication were more frequent among CIS patients prior to the oral examination (41 [28.3%] vs. 21 [14.4%]).

**Table 2.** Comparison of Oral Health Status and Medications between Patients with CIS and Stroke-Free Controls.

Characteristic	Classes	CIS Patient	Control	*P* Valuea
Number of missing teeth	0	108 (74.0)	114 (78.1)	0.267
	1–4	34 (23.3)	31 (21.2)	
	>4	4 (2.7)	1 (0.7)	
Mucosal lesions and changes	Yes (at least one)	23 (15.9)	19 (13)	0.617
Bifurcations	Yes (at least one)	25 (17.6)	16 (11.0)	0.151
Mobile teeth	Yes (at least one)	7 (4.9)	5 (3.4)	0.547
Cracked teeth	Yes (at least one)	20 (13.9)	10 (6.8)	0.078
Bleeding on probing (%)	<30	31 (21.8)	47 (32.4)	**0.027**
	30–50	75 (52.8)	73 (50.3)	
	>50	36 (25.4)	25 (17.2)	
Number of all periodontal pockets (≥4 mm)	0–2	36 (25.4)	66 (45.5)	**<0.001**
	3–10	65 (45.8)	64 (44.1)	
	11–30	30 (21.1)	14 (9.7)	
	>30	11 (7.7)	1 (0.7)	
Number of periodontal pockets (≥6 mm)	0	102 (71.8)	118 (81.4)	**0.023**
	1–2	28 (19.7)	22 (15.2)	
	3–5	6 (4.2)	3 (2.1)	
	>5	6 (4.2)	2 (1.4)	
Horizontal alveolar bone loss (%)	No bone loss	106 (73.1)	115 (79.3)	0.051
	<15	24 (16.6)	23 (15.9)	
	15–33	13 (9.0)	6 (4.1)	
	>33	2 (1.4)	1 (0.7)	
Antithrombotic medication^b^	None	1 (0.7)	143 (97.9)	**<0.001**
	ASA and/or dipyridamole	66 (45.2)	3 (2.1)	
	Clopidogrel	63 (43.2)	0 (0)	
	Anticoagulant	10 (6.8)	0 (0)	
	Two different antithrombotics	6 (4.1)	0 (0)	
Antihypertensive medication^b^	Yes	13 (8.9)	2 (1.4)	0.182
Statin medication^b^	Yes	109 (74.7)	8 (5.5)	**<0.001**
Antibiotic treatments^c^	Yes	41 (28.3)	21 (14.4)	**0.007**

ASA, low-dose acetylsalicylic acid; CIS, cryptogenic ischemic stroke.

a P < 0.05 are bolded.

b Current medication registered at 3 mo from stroke incident (CIS patients) or recruitment time (controls).

c Prescribed antibiotic treatment within 6 mo prior to oral examination.

### Oral Status

Table 2 displays a comparison of oral health characteristics and current medication between CIS patients and controls. Compared to controls, CIS patients had a higher bleeding on probing (BOP) index (median 41.5%, IQR 31.1%–50.4% vs. 35.1%, 27.6%–44.6%). The difference in alveolar bone loss was of borderline significance (*P* = 0.051), although “no bone loss” was less frequent in CIS patients than in controls (73.1% vs. 79.3%). CIS patients had a higher number of increased PPD (≥4 mm, *P* < 0.001; ≥6 mm, *P* = 0.023) and higher PIBI (median 6.0, IQR 2.3–13.0 vs. 3.0; 1.0–7.0; *P* < 0.001). The number of teeth was similar in CIS patients and controls (median 28, IQR 27–29 vs. 28, 28–29, *P* = 0.347). There was no significant difference in the prevalence of mucosal lesions (*P* = 0.617), bifurcations (*P* = 0.151), or mobile teeth (*P* = 0.547).

**Figure 1.**
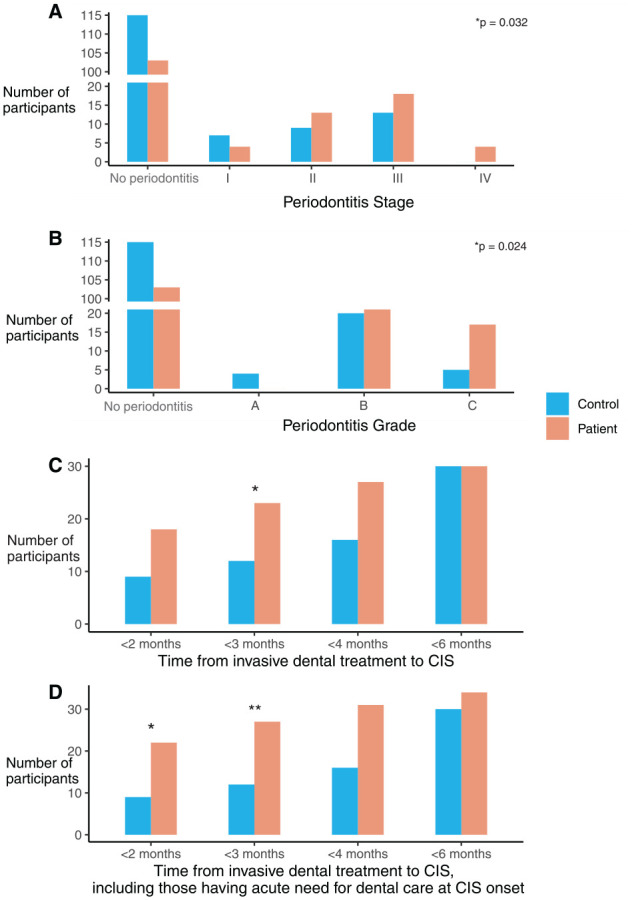
Periodontitis and recent invasive dental procedures in CIS patients and controls. Periodontitis stage (**A**) and grade (**B**) in patients with cryptogenic ischemic stroke (CIS) and age- and sex-matched stroke-free controls. *P* values in A and B were obtained from a Wilcoxon matched pairs signed rank test, which tests the null hypothesis that the median difference in periodontitis stage or grade between CIS patients and controls would be zero. Number of CIS patients and controls who underwent invasive dental treatments during the preceding 2 to 6 mo before CIS or recruitment (**C**) and including participants with acute dental infection who would have required dental treatment at the time of CIS onset (**D**). *P* values were obtained from conditional logistic regression adjusted for age. *P < 0.05, **P < 0.01.

A total of 39 (27.5%) CIS patients and 29 (20.1%) controls (*P* = 0.175) had periodontitis. We observed only stage I to III periodontitis among controls, whereas CIS patients also had the most severe form of the disease (stage IV). In addition, controls exhibited all grades of periodontitis, whereas CIS patients showed only putatively faster-progressing grades B and C (Fig. 1). Among the CIS patients, those who had periodontitis more often had moderate to severe than mild CIS (Appendix Fig. 1, Appendix Table 1, *P* = 0.030). Stage III to IV (11.2% vs. 30%) and grade C (23.3% vs. 8.6%) were the most frequent diagnoses of periodontitis among moderate to severe CIS compared to mild CIS. In a logistic regression model adjusted for age and sex, moderate to severe CIS was associated with having at least periodontitis stage II and grade C with an OR of 3.67 (95% CI, 1.177–11.203) and with PIBI >10 with an OR of 5.17 (95% CI, 1.50–21.9) (Appendix Table 2).

A total of 32 (21.9%) CIS patients and 30 (20.5%) controls had undergone invasive dental treatments during the preceding 6 mo before CIS or had an acute dental care need at the time of CIS/recruitment (Fig. 1). Most of the procedures in CIS patients (91.2%) occurred during the preceding 4 mo or they had an acute dental care need, whereas the corresponding percentage for controls was 53.3%. CIS patients experienced tooth extraction, endodontic treatment, fillings, and persisting dental infections more often than controls (Appendix Fig. 2).

### Laboratory Measures of Bacteremia

**Figure 2.**
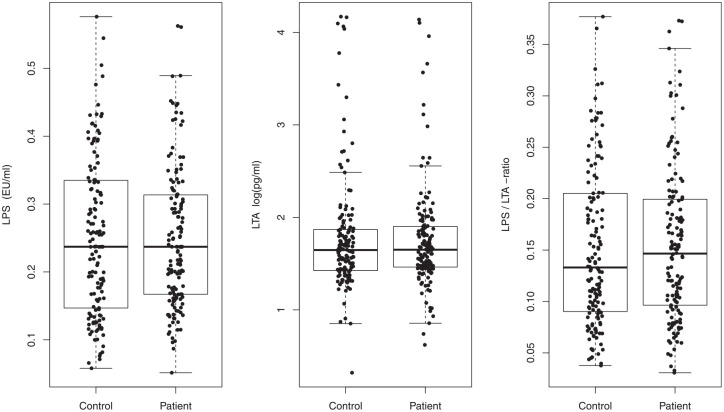
Serum lipotechoic acid (LTA) concentration and lipopolysaccharide (LPS) activity in cryptogenic ischemic stroke (CIS) patients and controls. Wilcoxon signed rank test for paired samples showed no difference between the groups.

LPS activity, LTA concentration, and their ratio (LPS/LTA) in serum were similar in CIS patients and controls (Fig. 2). Furthermore, LPS activity and LTA concentration were similar in different periodontitis stage and grade groups, while the LPS/LTA ratio showed an increasing significant trend toward more severe (*P* = 0.034) and faster-progressing (*P* = 0.022) periodontitis (Appendix Fig. 3). Time since invasive dental treatment did not correlate with LPS, LTA, or their ratio.

### Association between Periodontitis Parameters and Invasive Dental Procedures and CIS

In the fully adjusted conditional logistic regression models, having at least stage II and grade B periodontitis (*P* = 0.049) or having at least stage III and grade B periodontitis (*P* = 0.029) was associated with CIS (Table 3). Furthermore, having at least stage III and grade C periodontitis showed a strong association with CIS (*P* = 0.028). PIBI categories were associated with CIS in all models, with the strongest association found for PIBI >10 in model 3 (*P* < 0.001).

**Table 3.** Multivariable Conditional Regression Models on the Association between Periodontitis and Cryptogenic Ischemic Stroke in the Young.

Characteristic	CIS Patients	Controls	Model 1, OR (95% CI)	Model 2, OR (95% CI)	Model 3, OR (95% CI)
Periodontitis	*n* (%)				
No	103 (72.5)	115 (79.9)	Reference	Reference	Reference
Stage I	4 (2.8)	7 (4.9)	0.61 (0.17–2.21)	0.66 (0.15–2.85)	0.42 (0.08–2.27)
Stage II	13 (9.2)	9 (6.3)	1.36 (0.54–3.47)	1.52 (0.51–4.52)	1.43 (0.41–4.94)
Stages III–IV	22 (15.5)	13 (9.0)	2.35 (0.96–5.81)	2.64 (0.88–7.95)	2.67 (0.86–8.33)
Grade A	0 (0.0)	4 (2.8)	—	—	—
Grade B	22 (15.5)	20 (13.9)	1.30 (0.61–2.79)	1.39 (0.56–3.46)	1.36 (0.50–3.70)
Grade C	17 (12.0)	5 (3.5)	**4.02 (1.32–12.2)**	3.61 (1.00–13.0)	3.23 (0.85–12.3)
At least stage II + grade B	35 (24.6)	19 (13.2)	**2.19 (1.09–4.40)**	**2.33 (1.02–5.36)**	**2.45 (1.01–5.98)**
At least stage II + grade C	16 (11.3)	4 (2.8)	**4.85 (1.39–16.9)**	**4.59 (1.08–19.5)**	4.42 (0.98–20.0)
At least stage III + grade B	22 (15.5)	11 (7.6)	**3.30 (1.20–9.12)**	**3.63 (1.06–12.5)**	**4.11 (1.15–14.6)**
At least stage III + grade C	14 (9.9)	2 (1.4)	**6.78 (1.52–30.2)**	**7.15 (1.25–40.8)**	**7.48 (1.24–44.9)**
PIBI category					
0–2	36 (25.4)	65 (44.8)	Reference	Reference	Reference
3–10	63 (44.4)	64 (44.1)	**2.08 (1.18–3.68)**	**2.08 (1.07–4.04)**	**2.49 (1.21–5.13)**
>10	43 (30.3)	16 (11.0)	**8.68 (3.35–22.5)**	**8.77 (2.86–26.9)**	**10.5 (3.18–34.6)**
Invasive dental treatments					
Within 2 mo before	18 (12.3)	9 (6.2)	2.31 (0.98–5.44)	**3.39 (1.17–9.86)**	2.93 (0.97–8.92)
Including acute dental care need	22 (15.1)	9 (6.2)	**2.95 (1.28–6.80)**	**4.08 (1.43–11.7)**	**3.13 (1.06–9.19)**
Within 3 mo before	23 (15.8)	12 (8.2)	**2.14 (1.03–4.45)**	**2.98 (1.22–7.28)**	**2.54 (1.01–6.39)**
Including acute dental care need	27 (18.5)	12 (8.2)	**2.59 (1.26–5.32)**	**3.42 (1.42–8.25)**	**2.67 (1.09–6.57)**
Within 6 mo before	30 (20.1)	30 (20.1)	1.04 (0.59–1.84)	1.21 (0.61–2.40)	1.11 (0.53–2.30)
Including acute dental care need	34 (23.3)	30 (20.1)	1.23 (0.71–2.13)	1.38 (0.71–2.68)	1.19 (0.58–2.41)
Serum measurements	Mean (SD)				
LPS, EU/mL	0.26 (0.13)	0.26 (0.15)	0.62 (0.07–5.36)	0.38 (0.03–4.84)	0.09 (0.01–1.40)
LTA, log(pg/mL)	1.76 (0.56)	1.79 (0.64)	0.95 (0.65–1.39)	0.93 (0.59–1.45)	0.75 (0.47–1.21)
LPS/LTA ratio	0.16 (0.08)	0.16 (0.09)	0.91 (0.02–35.2)	1.00 (0.01–75.9)	0.29 (0.01–15.8)

Associations with *P* < 0.05 are bolded. Conditional logistic regression model. Variables presented in the table were analyzed in separate models. Model 1 is adjusted for age; model 2 was additionally adjusted for waist-to-hip ratio, smoking (never/ever), patent foramen ovale, and heavy alcohol consumption (yes/no); and model 3 was further adjusted for education level (low/high), regular dentist visits (regular/not regular), and hypertension status.

CI, confidence interval; CIS, cryptogenic ischemic stroke; LPS, lipopolysaccharide; LTA, lipoteichoic acid; OR, odds ratio; PIBI, periodontal inflammation burden index.

Recent invasive dental treatments, particularly those that occurred within 3 mo before CIS, were significantly associated with CIS (Table 3, Appendix Fig. 2). Invasive dental treatments occurring within 2 mo before CIS showed an association with CIS only in model 2 (*P* = 0.025), while the significance was suggestive in the fully adjusted model 3 (*P* = 0.058). However, invasive dental treatments combined with an acute dental care need prior to CIS strengthened the associations so that in the fully adjusted model, procedures in both 3-mo (*P* = 0.032) and 2-mo (*P* = 0.038) windows were significantly associated with CIS. When the period under consideration was widened to 6 mo before CIS, all the associations disappeared.

### Interaction Analyses

The association of periodontitis with CIS was not dependent on PFO status, whereas the association with invasive dental treatments was (*P* = 0.009 for interaction term for invasive treatments within 3 mo × PFO). In the subgroups based on PFO status, the association between CIS and invasive dental treatments was strong among those having PFO but not among the rest of population (Appendix Table 3). Other main parameters in the regression models did not show an interaction with PFO.

## Discussion

In our study, we found that young patients with CIS had a higher prevalence of a range of periodontal disease manifestations compared to age- and sex-matched stroke-free controls. Studies investigating stroke, and more specifically CIS, using the current classification of periodontitis have long been warranted. We found that having at least moderately severe and progressed periodontitis was associated with CIS independently from relevant confounding factors, and the strength of the association seemed to be dose dependent so that more severe periodontitis had a stronger association with CIS. Among the patients, stroke severity was also associated with worse periodontal condition. In addition to periodontitis, CIS patients had more often undergone recent invasive dental treatments or had persisting dental infections requiring acute dental treatment, thereby further linking oral inflammation with CIS in the young.

Our main findings comply with an earlier study by Grau et al. (2004), where the risk for CIS in a small group of all-aged patients (*n* = 53) was associated with a ≥4.5-mm clinical attachment loss. In that study, the association between attachment loss and CIS was independent of hypertension, diabetes, smoking, visits to a dentist, number of teeth, previous stroke, and father’s profession. In our study, the combined stage III–IV with grades B–C had a significant association with CIS independently from PFO, smoking, abdominal obesity, heavy alcohol consumption, or level of education. Further, PIBI, composed of periodontal probing depths, was highly significant in all our models. Notably, these 2 studies are, to our best knowledge, the only ones investigating periodontitis as a risk factor for CIS, whereas several studies have reported an association between periodontitis and cerebrovascular events with other etiologies.

Our results also align with those of a systematic review that demonstrated positive associations between periodontitis and all strokes among cohort studies in broader age groups (risk ratio, 1.88; 95% CI, 1.55–2.29; *P* < 0.001) and for ischemic stroke events in case-control studies (risk ratio, 2.72; 95% CI, 2.00–3.71; *P* < 0.001) (Fagundes et al. 2019). Stroke severity has not been previously reported to associate with periodontal parameters in young-onset CIS, as in the current study, but the association has been suggested in older participants (Slowik et al. 2010). More recent studies have also demonstrated associations between stroke and periodontitis (Lin et al. 2019; Lee et al. 2022), but evidence using the current classification of periodontitis applied in our study has been limited. We note that recent studies showing an association between periodontitis and ischemic stroke did not include education or socioeconomic level among the confounding factors (Lin et al. 2019; Lee et al. 2022). Our study used education as a measure of socioeconomic status, since along with occupational classes, it is a good predictor of health in Finnish epidemiological studies (Laaksonen et al. 2005). In addition, we included the information on self-reported regular dentist visits in the fully adjusted regression models as a proxy of oral health care habits.

Serum LPS and LTA were determined in our study to assess potential molecular pathways linking periodontitis and CIS. LPS and LTA are putative markers of bacterial translocation—an important mechanism likely linking oral inflammations and cardiovascular disease (Pussinen et al. 2022). However, serum LPS or LTA levels did not differ between our CIS patients and controls. Previous studies have reported elevated endotoxemia in cardiovascular disease events, including stroke (Pussinen et al. 2007; Klimiec et al. 2016), and recently even among a young-onset CIS population (Zheng et al. 2022). In the current population, samples were collected a few days after stroke incidence. At this point, CIS patients had started their antithrombotic medication, and many had received thrombolytic therapy as a treatment at the time of the event, which may have technically affected the measurements.

One possible, but not exclusive, source of LPS and LTA in systemic circulation is the oral cavity, particularly in dysbiotic diseases accompanied by impaired integrity of the oral epithelial barrier, such as in periodontitis (Liljestrand et al. 2017; Chidambaram et al. 2022; Pussinen et al. 2022). While LPS and LTA levels did not show differences between CIS patients and controls in our present study, the LPS/LTA ratio had a rising significant trend toward more progressed periodontitis. The shift in oral microbiota toward dysbiosis includes a change from Gram-positive-rich to more Gram-negative predominance of the oral microbiota in periodontitis (Chen et al. 2018), which could explain part of the circulatory LPS/LTA ratio pattern seen in the current study.

Dental care and regular dentist visits may reduce the risk of stroke related to oral health (Sen et al. 2018; Lin et al. 2019), but some dental care procedures, including tooth extractions and periodontal treatment, are invasive and may cause significant bacteremia (Lockhart et al. 2008). However, this was not seen in the measures of LPS or LTA in our study, which may be explained by the short half-time of these molecules in the circulation (Pussinen et al. 2022). In our study, the frequency of regular dentist visits (52.7% of CIS patients and 63.0% of controls) did not differ significantly. However, even after adjusting for common confounders, CIS patients had a higher frequency of recent invasive dental procedures, but the association was dependent on the PFO status. The classical mechanism linking PFO to CIS involves paradoxical embolism, allowing venous blood (e.g., from oral tissues) to bypass pulmonary filtration and enter cerebral circulation (Pristipino et al. 2019). This finding fits in the hypothesis that invasive dental treatments would have direct causality with CIS through bacteremia. Notably, the association with periodontitis remained independent of PFO, which in turn suggests that the mechanisms are more chronic in nature, such as low-grade inflammation or molecular mimicry (Pussinen et al. 2022).

A randomized controlled trial showed, already in 2007, that invasive dental treatment causes a decline in endothelial dysfunction, a potential trigger for thrombosis (Tuttolomondo et al. 2020), shortly after the treatment, but the effect is beneficial after 6 mo (Tonetti et al. 2007). Our result aligns with this finding, as we observed an association between CIS and invasive dental treatments within 2 or 3 mo before CIS, but once the period under consideration was extended to 6 mo before CIS, any negative effect disappeared.

Our study has strengths and limitations. To the best of our knowledge, this study is the largest study to date investigating the association of oral health with CIS in the young. Other strengths include rigorous periodontal measures: periodontal probing depth, bleeding on probing, horizontal alveolar bone loss, and using the latest classification of periodontitis stages and grades. Although we discovered strong associations among the studied parameters, it is worth noting that case-control studies tend to overestimate the effect strength. We managed to include almost all consecutive CIS patients treated in the participating centers during the recruitment period, but the number of individuals in some of the categories of the studied variables was relatively small, leading to a higher uncertainty of the effect sizes. In addition to education level, we did not have more robust information on the lifestyle or socioeconomic status. It is also possible that stroke event induced psychological and/or physical stress that has affected periodontal status before the clinical oral examination (Aoki et al. 2024). The oral examination was performed after a notable delay after a CIS event due to the guidelines, which may also have affected the oral status results.

In conclusion, our study demonstrates that periodontal inflammation and recent invasive dental procedures are associated with mechanisms in the development of CIS among young adults. The role of bacteremia as a mediator of this risk was not confirmed, and further studies are warranted to estimate the favorable effect of oral health on CIS incidence.

## Author Contributions

J. Leskelä, contributed to data acquisition, analysis, and interpretation, drafted the manuscript; J. Putaala, P.J. Pussinen, S. Paju, contributed to conception, design, data acquisition, analysis, and interpretation, critically revised the manuscript; N. Martinez-Majander, contributed to conception, design, data acquisition and interpretation, critically revised the manuscript; L. Tulkki, P. Ylikotila, R. Lautamäki, A. Saraste, S. Suihko, E. Könönen, J. Sinisalo, contributed to data acquisition, critically revised the manuscript; M. Manzoor, contributed to data interpretation, critically revised the manuscript; S. Zaric, contributed to design, critically revised the manuscript. All authors gave final approval and agree to be accountable for all aspects of the work.

## Supplemental Material

sj-docx-1-jdr-10.1177_00220345241232406 – Supplemental material for Periodontitis, Dental Procedures, and Young-Onset Cryptogenic Stroke Supplemental material, sj-docx-1-jdr-10.1177_00220345241232406 for Periodontitis, Dental Procedures, and Young-Onset Cryptogenic Stroke by J. Leskelä, J. Putaala, N. Martinez-Majander, L. Tulkki, M. Manzoor, S. Zaric, P. Ylikotila, R. Lautamäki, A. Saraste, S. Suihko, E. Könönen, J. Sinisalo, P.J. Pussinen and S. Paju in Journal of Dental Research
