# Supplementary material for: Periodontitis, Dental Procedures, and Young-Onset Cryptogenic Stroke
Source: J Dent Res. 2024 Apr 16;103(5):494–501. doi: 10.1177/00220345241232406 (PMC11047014; doi:10.1177/00220345241232406)
Supplement: sj-docx-1-jdr-10.1177_00220345241232406 – Supplemental material for Periodontitis, Dental Procedures, and Young-Onset Cryptogenic Stroke [file sj-docx-1-jdr-10.1177_00220345241232406.docx]

**Periodontitis, Dental Procedures, and Young-Onset Cryptogenic Stroke**

**Leskelä Jaakko, Putaala Jukka, Martinez-Majander Nicolas, Tulkki Lauri, Manzoor Muhammed, Zaric Svetislav, Ylikotila Pauli, Lautamäki Riikka, Saraste Antti, Suihko Satu, Könönen Eija, Sinisalo Juha, Pussinen Pirkko J, Paju Susanna**

**Supplementary Data**

**Supplementary Methods**

**Control recruitment**

Patients were matched for age and sex with stroke-free controls in a 1:1 ratio. A list of 20 potential controls per patient was randomly generated from the register of the Finnish Population Information System. Invitation letters were sent individually, one by one every two weeks, to these controls and if this approach did not yield a suitable and willing control participant, nonrelated proxies of patients or proxies of study personnel were enlisted.

**Risk factors and comorbidities**

All participants underwent a thorough structural interview conducted by the study personnel at the time of recruitment, and all available medical records were checked. Abdominal obesity was defined as a waist-to-hip ratio (WHR) of >0.85 in women and >0.9 in men. Heavy alcohol consumption was defined using an adapted version of the Alcohol Use Disorders Identification Test (AUDIT-C) questionnaire, where >5 points for women and >6 points for men were indicative of heavy alcohol use (WHO ASSIST Working Group 2002). Post-secondary or tertiary education were labeled as a high level of education. Patent foramen ovale (PFO) was diagnosed by a neurologist or cardiologist using transcranial Doppler ultrasound (“bubble study”) and/or transesophageal echocardiography (Tulkki et al. 2023). In patients, admission stroke severity was defined with the NIH Stroke Scale (NIHSS) and categorized as mild (NIHSS 0-4) and moderate-to-severe (NIHSS ≥5).

**Clinical oral examination**

The number of sites with probing pocket depth (PPD) of 4–5 mm and ≥6 mm, and bleeding on probing (BOP) were recorded from six sites per tooth. Further, bifurcations, mobile teeth, cracked teeth, and mucosal findings were recorded. PIBI was further categorized into three groups based on the index score: 0–2, 3–10, and >10.

**Questionnaire**

Questionnaire was filled in during the clinical examination visit. In the questionnaire, participants reported on antimicrobial (antibiotic) medication use in the preceding six months prior to the oral examination, regular dental attendance, and previous dental visits. Possible invasive dental procedures during the six months pre-stroke (or pre-recruitment for controls) and a symptomatic, acute need for dental care at the time of CIS onset (or recruitment time for controls) were registered. The subjects were asked to bring information on their previous dental visits if available.

**Laboratory analyses**

Serum samples were taken at the time of recruitment and stored at -70 °C. LPS activity was measured using an Endolisa assay (Biomerioux, France). Incubation time in the binding step was 18 hours at room temperature with continuous mild shaking. LTA concentration was measured using an ELISA assay (US Biological, Salem, Massachusetts) according to the manufacturer’s instructions. Distinct outliers (n=1 for LPS and n=0 for LTA) and missing samples or measurements (n=12 for LPS and n=0 for LTA) were replaced by the population median value.

**Statistical analysis**

We built multiple multivariate models through conditional logistic regression, gradually incorporating additional covariates. The fully adjusted model was adjusted for age, waist-to-hip ratio (WHR), heavy alcohol consumption, smoking, presence of a patent foramen ovale (PFO), education level, regular dentist visits, and hypertension status. We assessed the assumptions of a logistic regression model with no violations observed. Linearity was assessed with a visual inspection of residual plots of analogous linear models and collinearity with variance inflation factor analysis.

The interaction with PFO status was evaluated by introducing an interaction term into the fully adjusted conditional logistic regression models. Then, using two fully adjusted conditional logistic regression models – one with an interaction term, and one without it – we ran a likelihood ratio test to compare these two models. The interaction analysis was continued with the subgroups of subjects with or without PFO. We fitted unadjusted logistic regression models to in these subgroups to evaluate the difference in the association between CIS and recent invasive dental treatments in these groups.

Periodontitis and invasive dental treatments within 3 months before CIS event was analysed in the patients with mild (NIHSS score 0-4) and moderate to severe (NIHSS score ≥5) CIS. To analyse the parameters associated with stroke severity, we fitted a logistic regression model adjusted for age and sex.

**Supplementary Figure 1.** Periodontitis Stage (A) and Grade (B) in mild or moderate to severe stroke severity based on NIHSS score, 0–4 for mild and ≥5 for moderate to severe.


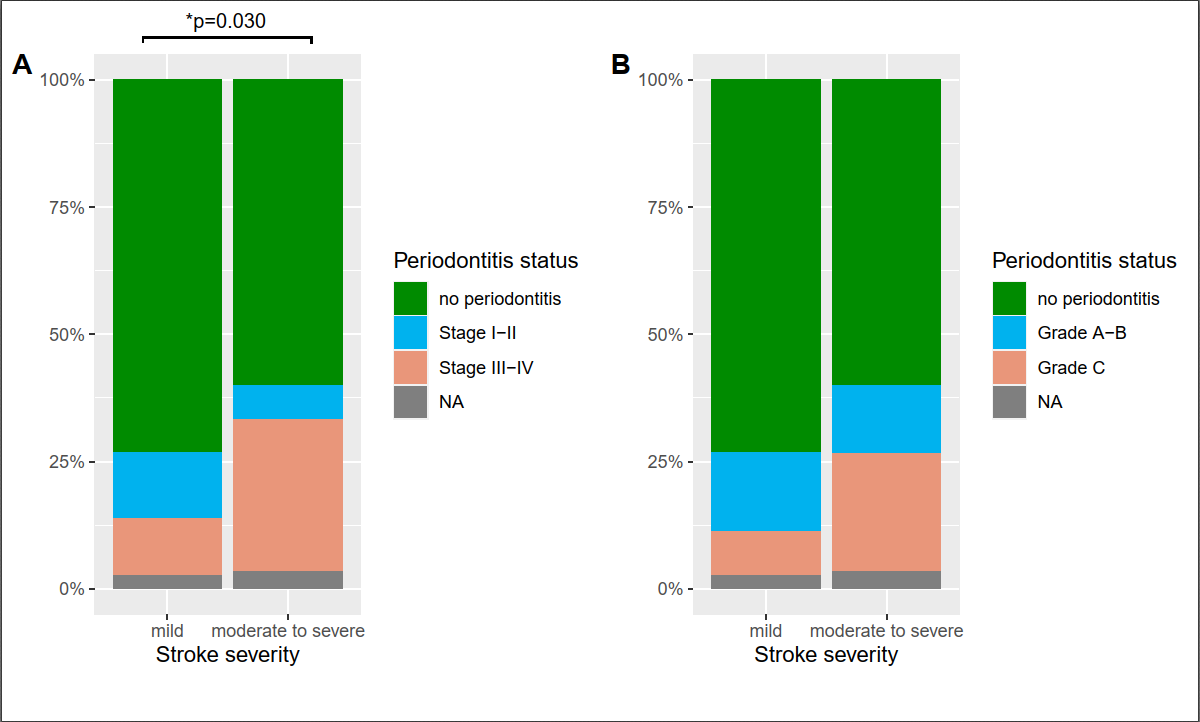


NIHSS, National Institutes of Health Stroke Scale

**Supplementary Figure 2.** Dental treatment categorization during the preceding three months before stroke/recruitment and persisting dental infections at the time of CIS onset/recruitment.


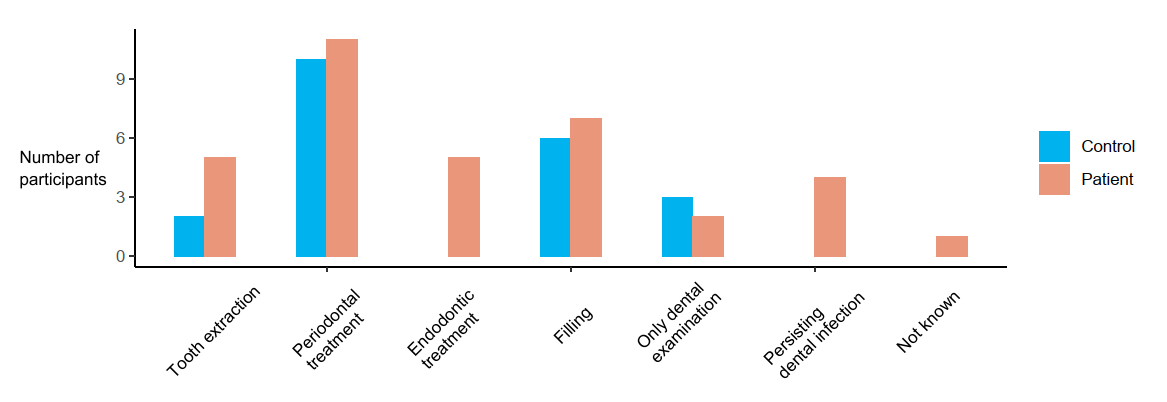


CIS, cryptogenic ischemic stroke

**Supplementary Figure 3.** Serum LPS activity and LTA concentration at different periodontitis stages and grade groups. Statistical trend testing was performed using the Jonckheere-Terpstra test.


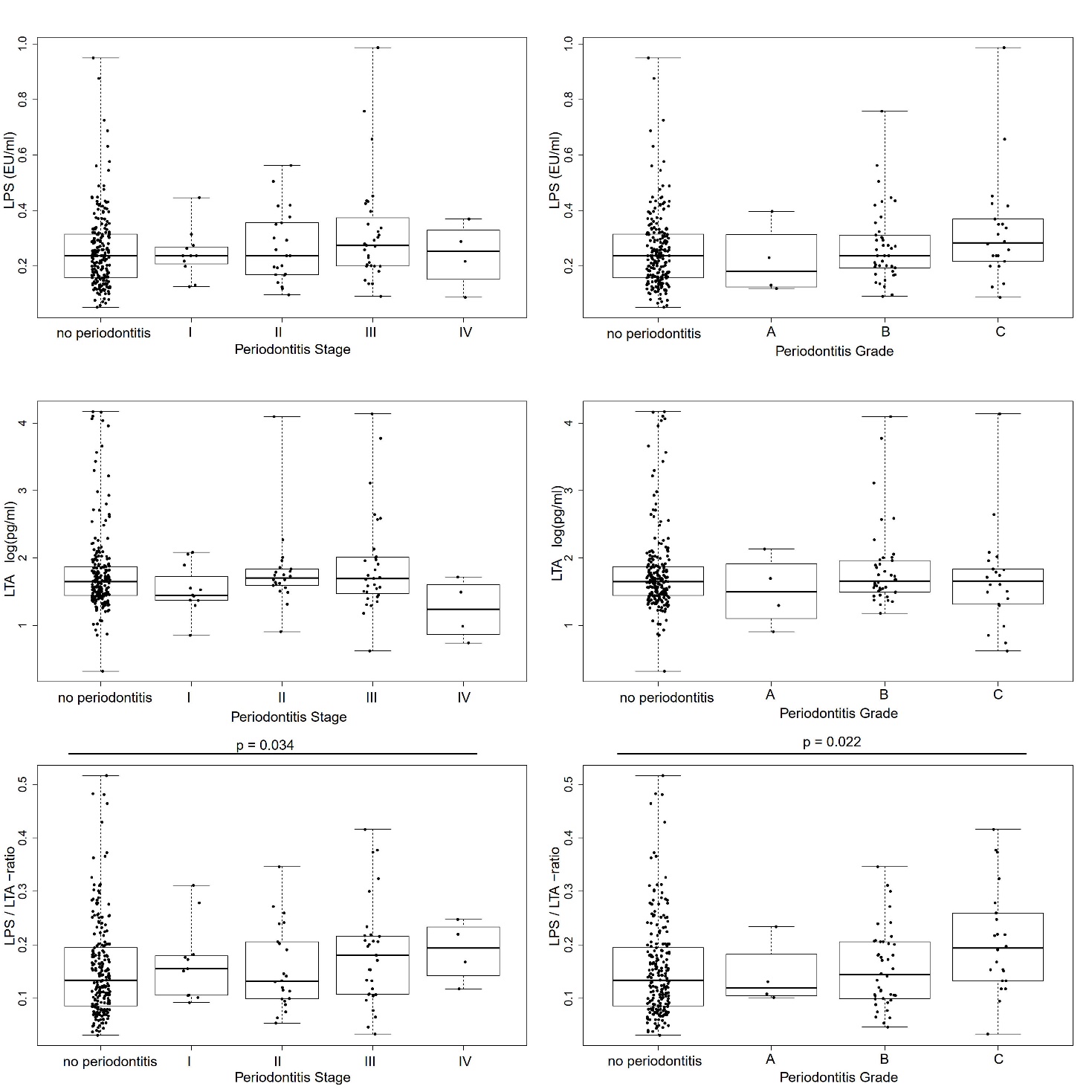


EU, endotoxin units; LPS, lipopolysaccharide; LTA, lipotechoic acid

**Supplementary Table 1.**

|  | **NIHSS category** | | p-value |
| --- | --- | --- | --- |
|  | mild, n (%) | moderate to severe, n (%) |  |
| total | 116 (100.0) | 30 (100.0) |  |
| missing periodontitis status | 3 (2.6) | 1 (3.3) |  |
| No periodontitis | 85 (73.3) | 18 (60.0) | – |
| Stage I–II | 15 (12.9) | 2 (6.6) | **0.030** |
| III–IV | 13 (11.2) | 9 (30.0) |  |
| Grade A | 0 (0.0) | 0 (0.0) | 0.077 |
| B | 18 (15.5) | 4 (13.3) |  |
| C | 10 (8.6) | 7 (23.3) |  |
| PIBI category 0–2 | 32 (27.6) | 4 (13.3) | 0.103 |
| 3–10 | 51 (44.0) | 12 (40.0) |  |
| >10 | 30 (25.9) | 13 (43.3) |  |
| Invasive dental treatment within 3 months before, including acute dental care need at incidence  yes | 22 (19.0) | 5 (16.7) | 0.980 |
| no | 94 (81.0) | 25 (83.3) |  |

P-value estimated using chi-square test, p-values below 0.05 significance threshold are in bold. NIHSS categories were as follows: mild, 0-4; moderate to severe, ≥5. NIHSS, National Institutes of Health Stroke Scale; PIBI, Periodontal inflammation burden index

**Supplementary Table 2. Logistic regression model for CIS severity.**

|  | **Mild CIS**  **NIHSS 0–4** | **Moderate-to-severe CIS**  **NIHSS ≥ 5** | **Logistic regression model** |
| --- | --- | --- | --- |
|  | **N (%)** | | **OR (CI)** |
| **Periodontitis** |  |  |  |
| no | 85 (73.3) | 18 (60.0) | Reference |
| stage I | 3 (2.6) | 1 (3.3) | 1.64 (0.08–14.9) |
| II | 12 (10.3) | 1 (3.3) | 0.47 (0.02–2.73) |
| III–IV | 13 (11.2) | 9 (30.0) | **4.94 (1.64–15.3)** |
| grade A | 0 (0.0) | 0 (0.0) |  |
| B | 18 (15.5) | 4 (13.3) | 1.34 (0.34–4.39) |
| C | 10 (8.6) | 7 (23.3) | **4.05 (1.27–12.7)** |
| at least stage II + grade B | 25 (21.6) | 10 (33.3) | 2.49 (0.94–6.58) |
| stage II + grade C | 9 (7.8) | 7 (23.3) | **4.50 (1.39–14.6)** |
| stage III + grade B | 13 (11.2) | 9 (30.0) | **4.98 (1.66–15.4)** |
| stage III + grade C | 7 (6.0) | 7 (23.3) | **6.43 (1.87–23.0)** |
| PIBI category |  |  |  |
| 0–2 | 32 (27.6) | 4 (13.3) | Reference |
| 3–10 | 51 (44.0) | 12 (40.0) | 2.01 (0.62–7.80) |
| >10 | 30 (25.9) | 13 (43.3) | **5.17 (1.50–21.9)** |
| **Invasive dental treatments** |  |  |  |
| within 2 months before | 16 (13.8) | 2 (6.7) | 0.48 (0.07–1.87) |
| including acute dental care need | 19 (16.4) | 3 (10.0) | 0.65 (0.14–2.13) |
| within 3 months before | 19 (16.4) | 4 (13.3) | 0.84 (0.23–2.52) |
| including acute dental care need | 22 (19.0) | 5 (16.7) | 0.96 (0.30–2.70) |

Logistic regression data are odds ratios (95% confidence interval) for moderate-to-severe CIS, associations with p<0.05 are bolded.

Logistic regression model for stroke severity. The models are adjusted for age and sex. Variables presented in the table were analysed in separate models. NIHSS categories were as follows: mild, 0-4; moderate to severe, ≥5. CIS, cryptogenic ischemic stroke; NIHSS, National Institutes of Health Stroke Scale; PIBI, Periodontal inflammation burden index

**Supplementary Table 3. Interaction analysis for PFO and invasive dental treatments**

|  | **Invasive dental treatment** | | **Unadjusted logistic model for CIS** |  |
| --- | --- | --- | --- | --- |
| Interaction factor | **within 3 months** | **no** | **OR (95% CI)** | **P-value for interaction** |
| **PFO** | N of CIS patients (controls) | |  | **0.009** |
| No | 4 (9) | 41 (77) | 0.835 (0.216–2.734) |  |
| Yes | 19 (2) | 82 (54) | **6.256 (****1.722–40.27)** |  |

Unadjusted logistic models for CIS were performed in subgroups based on PFO status. The fully adjusted models were adjusted for age, smoking, alcohol consumption, hypertension, PFO, regular dental examinations, and education.

CIS, cryptogenic ischemic stroke; OR, odds ratio; CI, Confidence interval; PFO, patent foramen ovale
